# An MSRE-Assisted Glycerol-Enhanced RPA-CRISPR/Cas12a Method for Methylation Detection

**DOI:** 10.3390/bios14120608

**Published:** 2024-12-12

**Authors:** Zhiquan Lu, Zilu Ye, Ping Li, Yike Jiang, Sanyang Han, Lan Ma

**Affiliations:** 1 Precision Medicine and Healthcare Research Center, Tsinghua-Berkeley Shenzhen Institute (TBSI), Tsinghua Shenzhen International Graduate School, Tsinghua University, University Town of Shenzhen, Nanshan District, Shenzhen 518055, China; luzq18@tsinghua.org.cn; 2 Institute of Biopharmaceutical and Health Engineering, Tsinghua Shenzhen International Graduate School, Tsinghua University, University Town of Shenzhen, Nanshan District, Shenzhen 518055, China; yezl19@tsinghua.org.cn; 3 Institute of Biomedical Health Technology and Engineering, Shenzhen Bay Laboratory, Shenzhen 518132, China; liping@szbl.ac.cn (P.L.); jiangyk@szbl.ac.cn (Y.J.)

**Keywords:** nasopharyngeal carcinoma (NPC), MSRE, RPA, CRISPR/Cas12a, fluorescence

## Abstract

Background: Nasopharyngeal carcinoma (NPC) is a malignant tumor with high prevalence in southern China. Aberrant DNA methylation, as a hallmark of cancer, is extensively present in NPC, the detection of which facilitates early diagnosis and prognostic improvement of NPC. Conventional methylation detection methods relying on bisulfite conversion have limitations such as time-consuming, complex processes and sample degradation; thus, a more rapid and efficient method is needed. Methods: We propose a novel DNA methylation assay based on methylation-sensitive restriction endonuclease (MSRE) HhaI digestion and Glycerol-enhanced recombinase polymerase amplification (RPA)-CRISPR/Cas12a detection (HGRC). MSRE has a fast digestion rate, and HhaI specifically cleaves unmethylated DNA at a specific locus, leaving the methylated target intact to trigger the downstream RPA-Cas12a detection step, generating a fluorescence signal. Moreover, the detection step was supplemented with glycerol for the separation of Cas12a-containing components and RPA- and template-containing components, which avoids over-consumption of the template and, thus, enhances the amplification efficiency and detection sensitivity. Results: The HGRC method exhibits excellent performance in the detection of a CNE2-specific methylation locus with a (limit of detection) LOD of 100 aM and a linear range of 100 aM to 100 fM. It also responds well to different methylation levels and is capable of distinguishing methylation levels as low as 0.1%. Moreover, this method can distinguish NPC cells from normal cells by detecting methylation in cellular genomes. This method provides a rapid and sensitive approach for NPC detection and also holds good application prospects for other cancers and diseases featuring DNA methylation as a biomarker.

## 1. Introduction

The incidence of nasopharyngeal carcinoma (NPC) exhibits a high prevalence in the southern regions of China [1]. Although Epstein–Barr Virus (EBV) antibody and EBV DNA testing can significantly improve the diagnosis of NPC, non-invasive screening using them alone may still lead to underdiagnosis with unsatisfactory clinical reliability [2,3]. Therefore, the development of new screening mechanisms for NPC holds significant value. Extensive evidence suggests that epigenetic abnormalities, including DNA methylation, are significantly implicated in the development of NPC [4,5,6]. DNA methylation serves as a crucial epigenetic mechanism for transcriptional regulation and plays a vital role in maintaining normal cellular functions [7,8]. During carcinogenesis, cytosines in CpG islands in the promoter regions of tumor suppressor genes acquire methylation, resulting in gene dysfunction or silencing [9,10]. Therefore, DNA methylation is regarded as a promising biomarker for early screening and diagnosis of NPC.

Conventional methods for screening DNA methylation events are mainly based on bisulfite conversion (BC), methyl CpG-binding domain (MBD) enrichment, and methylation-sensitive restriction endonuclease (MSRE) treatment [11,12,13,14,15]. Bisulfite converts cytosine to uracil while having no effect on methylated cytosine and is considered the gold standard due to its site-specific and genome-wide detection capability [16,17]. However, the BC is time-consuming and may lead to DNA degradation or incomplete conversion, resulting in false positivity [18]. MBD is a conserved structural domain in proteins that recognizes and binds methylated CpG in dsDNA [19]. MBD-enrichment-based methods are less susceptible to DNA damage but are limited by the non-specific binding of MBD proteins [20]. New avenues for DNA methylation detection methods are provided by MSRE, a class of endonucleases that are highly sensitive to base methylation and react with specific cleavage [21]. The cleavage of MSRE can be performed under milder conditions and in less time and can be easily combined with other methods for further signal enhancement [15,22,23,24]. The emergence of MSRE makes the pre-treatment of DNA simple and fast, but a relatively long processing time is still required to ensure complete digestion.

The clustered regularly interspaced short palindromic repeats (CRISPR)/Cas system is a powerful programmable gene-editing tool with wide applications in gene editing and nucleic acid detection [25,26]. Among them, CRISPR/Cas12a has attracted a lot of attention due to its simple operation and efficient trans-cleavage activity, which describes the non-specific cleavage of collateral ssDNA, providing a simple and efficient means of signal generation with the assistance of well-designed ssDNA probes [27]. Although the high efficiency of Cas12a enables amplification-free detection, the incorporation of nucleic acid amplification is necessary to achieve higher sensitivity [28]. Recombinase polymerase amplification (RPA) is a highly efficient isothermal amplification technique that obtains a large amount of product in a short period of time. Moreover, RPA has an optimal reaction temperature of 37 °C to 42 °C, making it highly compatible with CRISPR/Cas12a [29,30]. The one-pot detection of RPA and Cas12a allows for simultaneous reactions in the same tube, which achieves high sensitivity while avoiding contamination caused by multi-step reactions [31]. However, in conventional RPA-Cas12a one-pot detection, the excessive consumption of the template caused by the high efficiency of Cas12a limits further improvement in sensitivity. The use of appropriate additives such as sucrose and glycerol to physically separate the Cas12a-containing detection phase from the RPA- and template-containing amplification phases to a certain extent via density or viscosity was proved to be effective in controlling template over-consumption, which could further enhance the sensitivity [32,33].

Herein, we propose a novel HhaI digestion and Glycerol-enhanced RPA-CRISPR/Cas12a (HGRC) assay for DNA methylation. In this assay, we utilized the data collected in previous NPC methylation studies of our laboratory, that is, the differential methylation sites (DMS) of different human NPC cell lines and human nasopharyngeal epithelial cell lines obtained by methylation chip sequencing. According to the DMS data obtained, the NC_000006.12 (26020451C) locus of the H4C1 gene in transcript GRCh38.p14 exhibited significant hypermethylation in the CNE2 cell line of NPC and was selected as the target of detection due to the presence of protospacer adjacent motif (PAM) for Cas12a in its neighboring sequences. HhaI is employed to cleave unmethylated DNA during the digestion step, leaving structurally intact methylated DNA for subsequent RPA- Cas12a detection step, in which glycerol is added to improve amplification efficiency and sensitivity. By optimizing the conditions of digestion and detection step, this method achieves a faster and more sensitive detection of target DNA methylation. This method is also applied in genomes of CNE2 and normal epithelial cells for the detection of target methylation and verifies the high expression of it in CNE2.

## 2. Materials and Methods

**Preparation of methylated dsDNA.** The methylated and unmethylated ssDNAs were synthesized by Sangon, and in order to equalize the concentrations of methylated cytosine and corresponding dsDNA, the dsDNA was obtained by a single-extension PCR using methylated ssDNA as a template. The obtained product was subjected to a 2% agarose gel electrophoresis for 30 min at 120 V to separate dsDNA from impurities, followed by gel extraction to obtain target dsDNA with high purity. To avoid aerosol contamination, the loading operation in gel electrophoresis and extraction procedure of the unmethylated DNA was carried out first.

**HhaI digestion.** The HhaI digestion was carried out in a volume of 25 μL containing 20 μL target dsDNA with different concentrations, 10 U HhaI, 2.5 μL 10× rCutSmart buffer. The reaction mixture was incubated at 37 °C for 20 min and then terminated at 65 °C for 20 min. The product was stored at 4 °C.

**RPA and RPA-Cas12a detection.** RPA was conducted using the ERA kit manufactured by GenDx Biotech (Suzhou, China), consisting of three components: activator, solubilizer, and lyophilized powder of enzymes, which was divided into small portions and stored in 200 μL transparent PCR tubes. RPA was performed in a 50 µL reaction system in which 2 µL of activator was added to the inner cap of the tube, 20 µL solubilizer and 500 nM forward and reverse primer, 6.6 µL template with desired concentration was added to the bottom of the tube, comprising the remaining 48 µL volume. RPA was triggered by brief centrifugation for a few seconds, then shaken for 3 s on a vortex oscillator and incubated at 37 °C for 5 min, and the reaction was terminated by adding 10 μL of Proteinase K (10 mg/mL) and held at 58 °C for 10 min. The product was stored at 4 °C.

The RPA-Cas12a detection was conducted in a 60 µL system consisting of three components: Component A, Component B, and activator. Component B was added to the bottom of tube with a total volume of 37.6 µL, in which lyophilized powder of enzymes was dissolved, containing 24 µL solubilizer, forward and reverse primers with final concentration of 500 nM, 4 µL 10× NEBuffer 2.1, 6.6 μL target template with desired concentrations and DEPC-treated water. Component A was added to the inner wall of the tube without direct contact with Component B, with a total volume of 20 μL, consisting of 2 μL 10× NEBuffer 2.1, 60 U nuclease inhibitor, LbCas12a and crRNA with final concentration of 100 nM and 150 nM, respectively, and DEPC-treated water. A 2.4 μL activator was added to the inner cap of tube. The reaction was activated by a brief centrifugation for 3 s and shaking on a vortex oscillator for 3 s and incubated at 37 °C for a specific period of time, followed by holding at 65 °C for 10 min to terminate the reaction.

The reagents, apparatus, and detailed protocols of dsDNA preparation, cell culturing, genome extraction, and electrophoresis are presented in Supplementary Information.

## 3. Results and Discussion

- Working principle of HGRC

**Figure 1:**
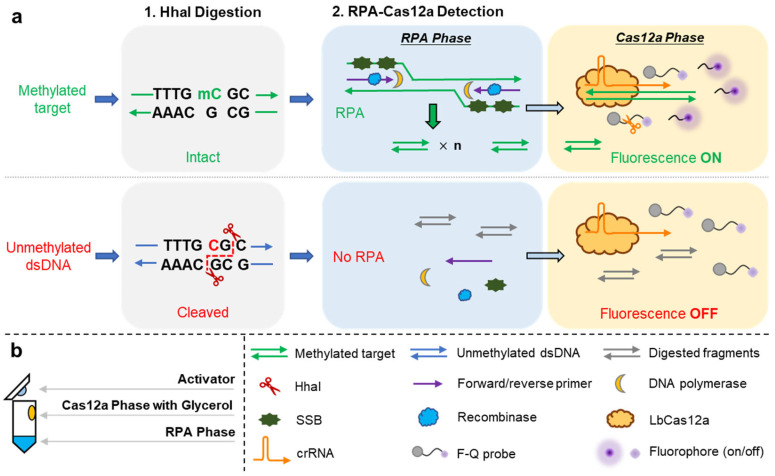
Schematic illustration of the HGRC system. (**a**) Workflow of the HGRC system; (**b**) schematic illustration of glycerol-enhanced detection step.

The working principle of HGRC is depicted in Figure 1. The proposed assay consists of two steps: digestion and detection. As depicted in Figure 1, the cleavage site of HhaI is unmethylated CGC↓G, where the arrow indicates the cleavage site, and no cleavage is conducted when methylated cytosine is present. In this method, the PAM of Cas12a is located upstream of the target methylation locus, so the unmethylated DNA after HhaI digestion will not be recognized by CRISPR RNA (crRNA) due to loss of PAM or spacer, which in turn fails to activate trans-cleavage in subsequent reaction. In contrast, the methylated target can be spared from cleavage and remain intact in the subsequent detection step. Moreover, there is no PAM or spacer in the forward or reverse primers, avoiding false positives caused by the primers themselves. In the detection step, glycerol is added to the Cas12a phase to separate it from the RPA phase by its high viscosity, thus allowing for a more adequate process of RPA, leading to increased sensitivity. In addition, to avoid premature mixing of the phases, the RPA phase, the Cas12a phase, and the RPA activator are added to the bottom, the inner wall, and the inside of the cap of the PCR tubes, respectively, without direct contact, and the detection is initiated by brief centrifugation. In summary, for the unmethylated target, no fluorescence signal will be generated during detection, while for the methylated target, a substantial fluorescence signal will be generated, and the target could be quantified by characterizing the intensity of the fluorescence signal.

- Analysis of HhaI digestion

**Figure 2:**
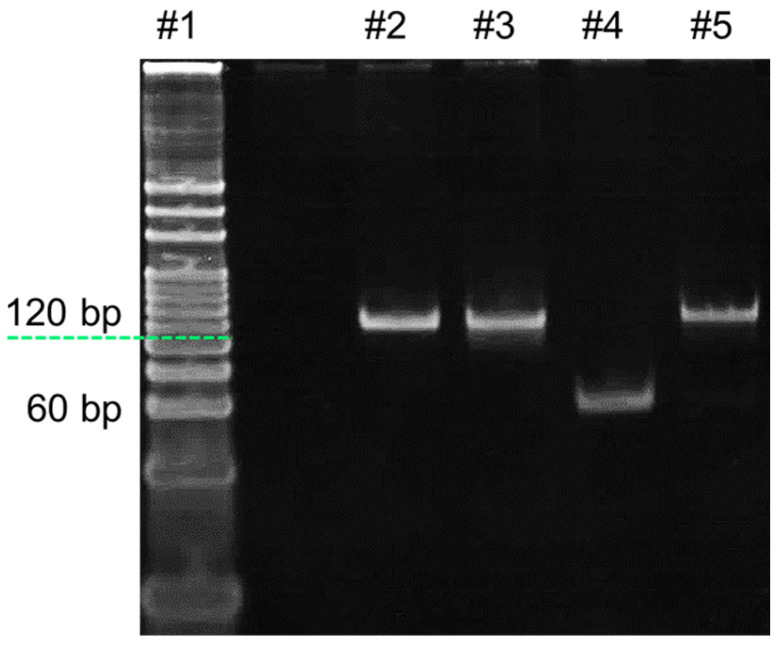
An 8% PAGE analysis of the products in the HhaI digestion. Lane 1: 20 bp DNA ladder; lane 2: Unmethylated dsDNA (10 nM); lane 3: Methylated target (10 nM); lane 4: Unmethylated dsDNA after HhaI digestion; lane 5: Methylated target after HhaI digestion.

The feasibility of the HhaI digestion process was analyzed by 8% PAGE, and the results are presented in Figure 2. Lanes 2 and 3 represent the unmethylated and methylated target dsDNA, with a length of 122 bp, while lanes 4 and 5 represent the fragments of unmethylated and methylated dsDNA after HhaI digestion. By comparing lane 4 and lane 5, it can be noticed that the mobility of the bands in lane 4 is significantly larger, which is due to the shorter length of the product. According to the DNA ladder, it can be further determined that the size of the product in lane 4 is about 60 bp, which is consistent with the template design. On the other hand, the size and mobility of the methylated target remained unchanged, indicating that it escaped from HhaI digestion. Taking the above evidence together, it can be concluded that the unmethylated dsDNA was successfully digested by HhaI.

- Analysis of RPA

**Figure 3:**
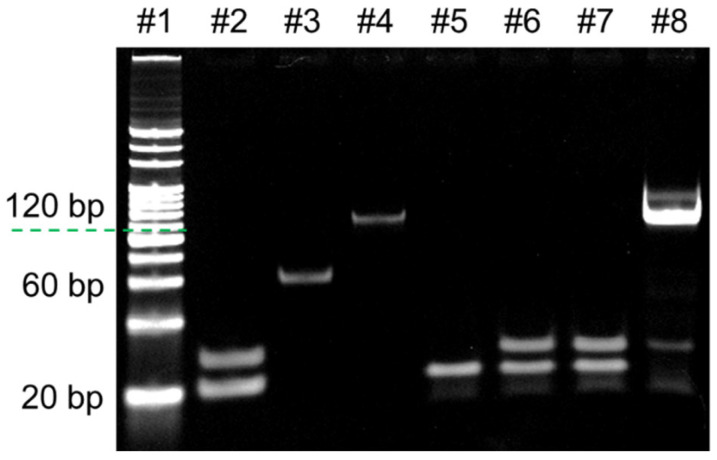
An 8% PAGE analysis of the products in RPA. Lane 1: 20 bp DNA ladder; lane 2: Forward and reverse primers (500 nM for each); lane 3–4: HhaI products of unmethylated and methylated target (10 nM), respectively; lane 5–6: RPA blank control with only reverse primer and both primers (500 nM), respectively; lane 7–8: RPA products of HhaI digested unmethylated and methylated targets (1 nM), respectively.

An 8% PAGE was also used to analyze the RPA product, and the results are shown in Figure 3. In RPA, the intact template and primers of both sides are required for exponential amplification. Bands in lanes 2–4 of Figure 3 represent the initial state of primers, unmethylated and methylated products of HhaI digestion, respectively. As depicted in lanes 5–7, the presence of bands representing primers and the absence of bands representing amplification products are observed, indicating that RPA could not be successfully performed only by primers and truncated template without the participation of the intact template. On the other hand, there is a distinct band at about 120 bp presented in lane 8, which is the same size as the template in lane 4, representing the amplification product, which indicates that RPA was successfully conducted in the presence of methylated product.

- Feasibility of HGRC

**Figure 4:**
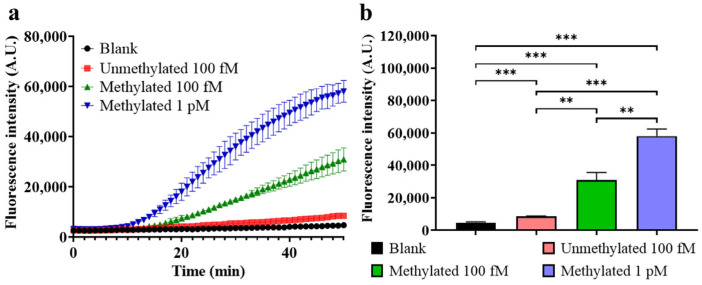
Feasibility test of HGRC. (**a**) Real-time fluorescence intensity of each group; (**b**) endpoint fluorescence intensity of each group after a 50 min reaction. (Blank control: DEPC-treated water, glycerol concentration 10% (*v*/*v*), HhaI concentration 0.4 U/μL, HhaI digestion time 15 min, probe concentration 400 nM. Mean ± s.d., *n* = 3 technical replicates. Variance was calculated using Student’s *t*-test, ** represents *p* < 0.01, *** represents *p* < 0.001.).

To further validate the feasibility of this method, the complete HGRC method was conducted for DEPC water, unmethylated target, or methylated target with different concentrations, and the real-time fluorescence signals and the endpoint fluorescence intensity after a 50 min reaction were recorded, and the results are shown in Figure 4. As depicted in Figure 4a, the fluorescence generation rate in the blank control is the lowest, followed by the unmethylated group, and the methylated groups have a larger fluorescence generation rate, which is positively correlated with the concentration. The comparison of the endpoint fluorescence intensity in Figure 4b demonstrates the difference between the groups more intuitively, indicating that HGRC is capable of distinguishing methylated targets from unmethylated dsDNA and blank control. Notably, the unmethylated group still produces some fluorescence, making it distinguishable from blank control, which may be due to incomplete digestion of HhaI. These intact unmethylated dsDNAs that evaded HhaI cleavage act as false positives and produce a large amount of fluorescence signal in the detection step; hence, the optimization of the HhaI digestion process is necessary to reduce false positive, which could be achieved by increasing HhaI dosage, extending the digestion time, and reducing the content of templates.

- Optimization of HGRC

To achieve the best performance of the HGRC method, several important reaction parameters were optimized, aiming at two aspects: one is to improve the reaction efficiency, and the other one is to reduce the background of detection. Considering the high rate of the positive groups and the incomplete digestion of the negative group, the total amount of DNA involved was reduced to 100 fM to ensure a high rate of methylated DNA as well as to accelerate the digestion of non-methylated DNA.

**Figure 5:**
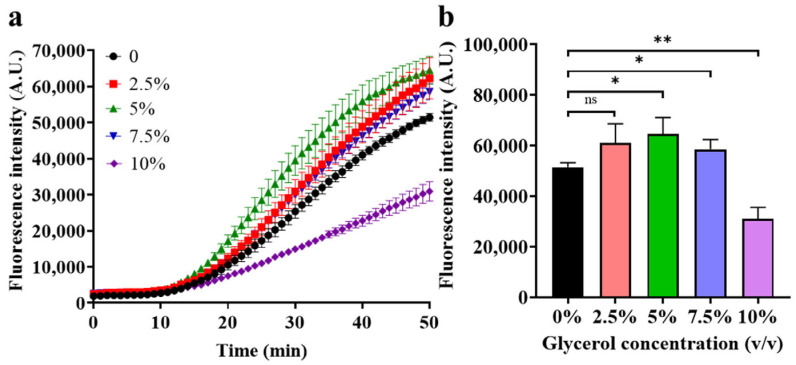
Optimization of glycerol concentration. (**a**) Real-time fluorescence intensity at different glycerol concentrations; (**b**) Endpoint fluorescence intensity after a 50 min reaction. (Methylated target concentration 100 fM, HhaI concentration 0.4 U/μL, HhaI digestion time 15 min, probe concentration 400 nM. Mean ± s.d., *n* = 3 technical replicates, variance was calculated using student’s *t*-test, * represents *p* < 0.05, ** represents *p* < 0.01, ns represents non-significant).

Firstly, the concentration of glycerol was optimized to enhance the efficiency of detection, and the results are shown in Figure 5. As depicted in Figure 5a, when the glycerol concentration is less than 5%, the fluorescence generation rate is positively correlated with the glycerol concentration, which indicates that the phase separation effect introduced by glycerol enables a more acceptable amplification and, therefore, enhances the fluorescence signal. However, when the glycerol concentration exceeds 5%, the fluorescence generation rate decreases with the increase in glycerol, which is due to the increased resistance of diffusion of the RPA products to the Cas12a phase and, therefore, weakens the fluorescence signal. A more intuitive comparison is given in Figure 5b, demonstrating that a glycerol concentration of 5% has the best performance. A similar glycerol concentration optimization experiment was performed on target methylation at a concentration of 1 pM, again indicating that 5% was the optimal glycerol concentration, from which a more significant kinetic difference can be observed, as shown in Figure S1.

**Figure 6:**
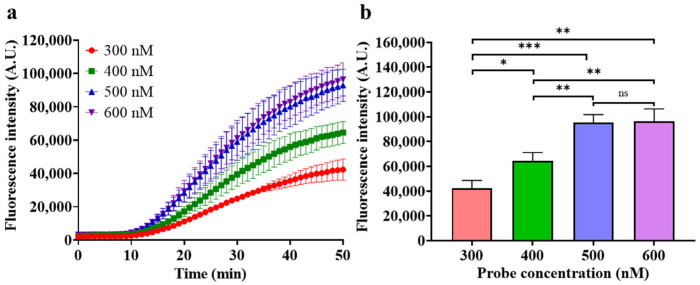
Optimization of probe concentration. (**a**) Real-time fluorescence intensity of different probe concentration; (**b**) endpoint fluorescence intensity of different probe concentrations after a 50 min reaction. (Target concentration 100 fM, glycerol concentration 5%, HhaI concentration 0.4 U/μL, HhaI digestion time 15 min, probe concentration 400 nM. Mean ± s.d., *n* = 3 technical replicates, variance was calculated using Student’s *t*-test, * represents *p* < 0.05, ** represents *p* < 0.01, *** represents *p* < 0.001, ns represents non-significant).

Then, the concentration of the F–Q probe was also optimized for optimal fluorescence generation efficiency, and the results are shown in Figure 6.

As depicted in Figure 6a, the fluorescence generation rate is positively correlated with probe concentration, and the increase slows when the concentration exceeds 500 nM. In the endpoint fluorescence intensity plot of Figure 6b, it can be more intuitively observed that the further increase in fluorescence intensity is very limited when the probe concentration exceeds 500 nM. Therefore, 500 nM was selected as the optimal concentration of the F–Q probe.

**Figure 7:**
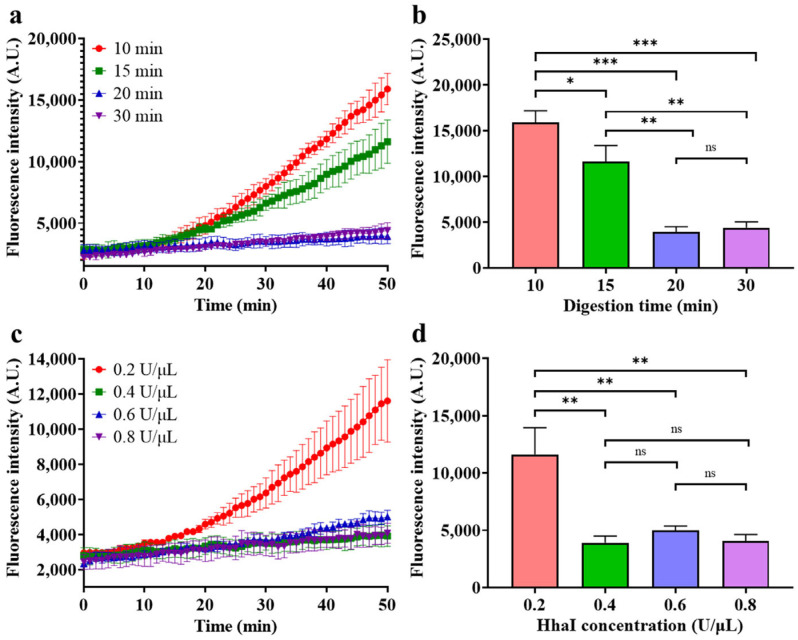
Optimization of HhaI digestion. (**a**) Real-time fluorescence intensity of different digestion time groups; (**b**) endpoint fluorescence intensity of different digestion time groups after a 50 min reaction; (**c**) real-time fluorescence intensity of different HhaI concentration groups; (**d**) endpoint fluorescence intensity of different HhaI concentration groups. (Target concentration 100 fM, glycerol concentration 5%, probe concentration 500 nM, HhaI concentration 0.4 U/μL for (**a**,**b**), HhaI digestion time 15 min for (**c**,**d**). Mean ± s.d., *n* = 3 technical replicates, variance was calculated using Student’s *t*-test, * represents *p* < 0.05, ** represents *p* < 0.01, *** represents *p* < 0.001, ns represents non-significant).

After determining the conditions for optimal fluorescence generation efficiency, the parameters of HhaI digestion, including HhaI concentration and time of digestion, were also optimized under these conditions, and the results are shown in Figure 7.

As depicted in Figure 7a, the fluorescence generation rate is negatively correlated with the digestion time when digestion time is below 20 min and no longer decreases when the digestion time exceeds 20 min, indicating complete digestion of unmethylated dsDNA after 20 min. The endpoint fluorescence intensity plots in Figure 7b intuitively illustrate that for dsDNA with a total amount of 100 fM, a 20 min digestion time is sufficient at an HhaI concentration of 0.4 U/μL. Then, the concentration of HhaI was further optimized, and the results are shown in Figure 7c,d. As depicted in Figure 7c,d, when the concentration of HhaI is 0.2 U/μL, some fluorescence is generated during the detection, which is caused by incomplete digestion, whereas the fluorescence intensity no longer decreases when the concentration of HhaI exceeds 0.4 U/μL, indicating complete digestion of unmethylated dsDNA. Therefore, 0.4 U/μL was selected as the optimal HhaI concentration.

- Sensitivity

**Figure 8:**
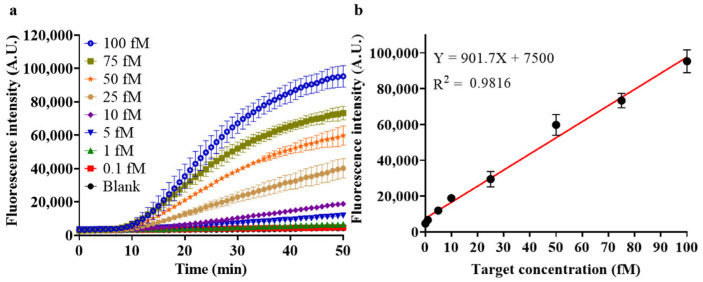
Detection of target methylation with different concentrations under optimal conditions. (**a**) Real-time Fluorescence intensity of detection; (**b**) fluorescence intensity—concentration linear relationship at the end of 25 min reaction. (Mean ± s.d., *n* = 3 technical replicates).

Under optimal conditions, quantitative detection of target methylation at different concentrations was performed, and the results are shown in Figure 8. As depicted in Figure 8a, the fluorescence generation rate in the detection step increases along with the increase of target concentration from 0.1 fM to 100 fM. Figure 8b shows the changes in endpoint fluorescence intensity (I) for detecting target methylation at different concentrations (C), and the equation I = 901.7C + 7500 is obtained after linear fitting, with an R^2^ of 0.9816, indicating a good linear relationship. The linear relationship at lower concentrations is presented in a zoomed-in plot in Figure S2. The comparison of 100 aM target and blank control by HGRC is also given in Figure S3, which indicates that HGRC is capable of detecting target methylation at a concentration as low as 100 aM.

A comparison of this method with previous methods is shown in Table 1, which demonstrates that compared to the conventional BC- and MBD-based methods, the introduction of HhaI significantly shortens the time-consuming pre-treatment of methylated DNA and, therefore, has a great advantage in terms of time-consumption. Meanwhile, with the aid of glycerol, this method presents both a shorter detection time and a high sensitivity. In addition, due to the flexibility of RPA primer design, this method does not strictly require the composition of the target sequence and is widely adaptable.

However, constrained by the requirement of complete digestion, the linear range of this method is limited to a certain extent, and the discovery of new and more efficient MSREs, as well as an extended digestion time, will contribute to improving the performance of this system further.

- Detection of different methylation level

**Figure 9:**
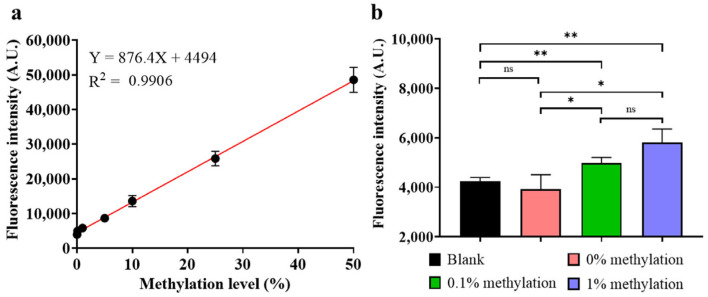
Detection of different methylation levels. (**a**) Relationship between endpoint fluorescence intensity and different methylation levels; (**b**) fluorescence intensity at the end of 25 min reaction with different miRNAs. (Mean ± s.d., *n* = 3 technical replicates, variance was calculated using Student’s *t*-test, * represents *p* < 0.05, ** represents *p* < 0.01, ns represents non-significant).

In practice, the target locus is usually in a coexisting state of demethylation and methylation, and the level of methylation varies. To match the practical scenario, the target locus under different methylation levels was also detected using HGRC. A mixture was prepared using unmethylated and methylated dsDNA with a total concentration of 100 fM, and the methylation level (L) was calculated by the formula: L = 100% × M/(M + U), where M and U represent methylated and unmethylated dsDNA, respectively. As depicted in Figure 9a, the relationship between fluorescence intensity (I) and methylation level (L) can be obtained by linear fitting, as described by the equation I = 876.4L + 4494, with an R^2^ of 0.9906, indicating a good linear relationship. In addition, as shown in Figure 9b, using DEPC-treated water and 0% methylated dsDNA as a blank and negative control, respectively, it can be observed that HGRC can successfully distinguish methylation levels as low as 0.1%. The response to different methylation levels further improved the practical value of this method.

- Methylation detection in cellular genomes

**Figure 10:**
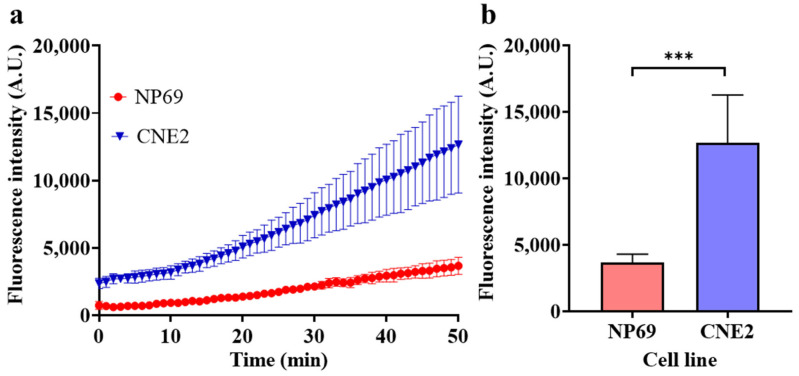
Methylation detection in genomes of different cell lines. (**a**) Real-time fluorescence intensity of methylation detection for each genome; (**b**) endpoint fluorescence intensities after a 50 min methylation detection for each genome by HGRC. (Mean ± s.d., *n* = 9, variance was calculated using Student’s *t*-test, *** represents *p* < 0.001).

To verify the DMS in NPC, a human NPC cell line CNE2 and human nasopharyngeal epithelial cell line NP69 were cultured, and their genomes were extracted and subjected to methylation detection by HGRC. In consideration of the heterogeneity of the tumor cells, for each cell line, cells were harvested for three samples weekly, for a total of nine sets of samples after three weeks. The genomes of the harvested cells were extracted and initially quantified, and the concentration was uniformly adjusted to 75 ng/μL for subsequent methylation detection. The genomes were then subjected to methylation detection using the HGRC system, and the results are shown in Figure 10.

As depicted in Figure 10a, the fluorescence generation rate of CNE2 is significantly larger than that of NP69, indicating a high level of target methylation in it. As depicted in Figure 10b, the endpoint fluorescence intensity of the CNE2 group is significantly larger than that of the NP69 group, indicating a large amount of target methylation in the CNE2 genome. In addition, it can also be found from Figure 10 that the variance of fluorescence intensity in the CNE2 group is much larger than that of NP69, which may be due to the heterogeneity of cancer. The methylation detection in genomes verified the hypermethylation of the target locus and indicated that HGRC is capable of distinguishing NPC cells from normal nasopharyngeal epithelial cells, providing a new method for screening and diagnosis of NPC.

The detection of cellular genomes illustrates the application potential of this method in the diagnosis of NPC and other diseases that use methylation as a biomarker. Due to the limitation of sample types, it is currently validated only in cell line genomes, and the application for clinical samples or further, non-invasively obtained clinical samples would increase the potential of this method in the clinical diagnosis of diseases even further.

## 4. Conclusions

Early screening and diagnosis of NPC is an important aid in improving prognosis, and the detection of DMS provides a promising entry point for it. In this work, the HGRC detection system for methylation in NPC was established with the assistance of HhaI digestion and glycerol-enhanced RPA-Cas12a detection. The proposed system shortens the reaction time, improves the sensitivity, and can detect methylation levels as low as 100 aM with good linearity in the concentration range of 0.1–100 fM. This method also responds well to different levels of methylation and can distinguish methylation levels as low as 0.1% in normal DNA. Moreover, the HGRC method can be applied for DMS detection in cellular genomes and distinguish NPC cells from normal human nasopharyngeal epithelial cells. In addition, due to the programmability of CRISPR, this method can be used for the detection of DMS in other diseases, contributing to screening and diagnosis.

## Figures and Tables

**Table 1:** The comparison of HGRC with previous methods.

Target	Detection Strategy	Method of Detection	Analysis Time	Limit of Detection (LOD)	Reference
Customized target	Antibody + GO	DPV	~5 h	1 fM	[34]
miR200b P2 promoter	Bisulfite + PCR	SERS	>24 h	0.5 pM	[35]
TIMP-3 gene	Bisulfite + LCR	Colorimetric	~90 min	0.01 fM	[36]
Septin 9 gene	GlaI + EXPAR	fluorescence	~2 h	200 aM	[15]
Customized target	HpaII + HCR	CV	>6 h	0.93 aM	[22]
Customized target	HpaII + UCNPs + AuNR	Fluorescence	~2 h	7 pM	[37]
Septin9 gene	GlaI + DC-SDA + Cas12a	Fluorescence	>3 h	128 fM	[24]
SEPT9 gene	BstUI + HhaI + RPA + Cas13a	Fluorescence	~90 min	86.4 aM	[38]
CNE2-specific methylation	HhaI + glycerol + RPA-Cas12a	Fluorescence	70 min	100 aM	This work

Abbreviations: GO, graphene oxide; DPV, differential pulse voltammetry; SERS, surface-enhanced Raman scattering; LCR, ligase chain reaction; EXPAR, exponential amplification reaction; HCR, hybridization chain reaction; CV, cyclic voltammetry; UCNPs, upconversion nanoparticles; DC-SDA, double cascaded strand displacement amplification.

## Data Availability

The original contributions presented in this study are included in the article/Supplementary Materials. Further inquiries can be directed to the corresponding authors.

## Supplementary Materials

The following supporting information can be downloaded at: https://www.mdpi.com/article/10.3390/bios14120608/s1.
