# Supplementary material for: An MSRE-Assisted Glycerol-Enhanced RPA-CRISPR/Cas12a Method for Methylation Detection
Source: Biosensors (Basel). 2024 Dec 12;14(12):608. doi: 10.3390/bios14120608 (PMC11674872; doi:10.3390/bios14120608)
Supplement: Supplementary file 1 [file biosensors-14-00608-s001.zip › biosensors-3329722-supplementary.pdf]

Supplementary Information

An MSRE-Assisted Glycerol-Enhanced RPA-CRISPR/Cas12a Method for Methylation Detection

1. Experimental Methods

Reagents and apparatus

HhaI was purchased from New England BioLabs, LbCas12a and F-Q probe were purchased from Bio-lifesci (Guangzhou, China), Gel-red nucleic acid gel stain, RNase inhibitor were purchased from Beyotime Biotech (Jiangsu, China), 20 bp DNA Ladder was purchased from Takara Bio (Beijing, China), 30% Acr-bis (29:1) was purchased from Servicebio (Wuhan, China), pancreatin was purchased from Yeasen Biotech (Shanghai, China), PCR was conducted via Phanta Flash Master Mix kit manufactured by Vazyme (Nanjing, China), agarose was purchased from Biowest, the gel extraction kit was purchased from Omega Bio-Tek, Proteinase K (10 mg/mL) and the cells genomic DNA extraction kit was purchased from Solarbio (Beijing, China), RPA was performed using the ERA kit purchased from GenDx Biotech (Suzhou, China). Cell culture medium RPMI1640, Penicillin-streptomycin (PS) and fetal bovine serum (FBS) were purchased from Thermal Fisher, other chemical reagents were purchased from Macklin (Shanghai, China). All the primers were synthesized by Sangon (Shanghai, China) and the sequences were shown in Supporting information. The human nasopharyngeal epithelial cell line NP69 and human NPC cell line CNE2 were obtained from Bena Culture Collection (Henan, China).

The electrophoresis results were visualized on a WB-9413B gel imaging analyzer (Liuyi biotech, Beijing). The extracted genomes were quantified by a NanoDrop One UV-Vis microspectrophotometer (Thermal Fisher). All the nucleic acid reactions were carried out in a ProFlex^{TM} 3x32 thermal cycler (Thermal Fisher), the fluorescence

spectra were recorded by a qTOWER^3 qPCR detecting system (Analytik Jena AG).

Protocols of buffer solutions

Cell freezing medium: 90% fetal bovine serum (FBS), 10% dimethyl sulfoxide (DMSO).

1× PBS buffer: 137 mM NaCl, 10 mM Na_2HPO_4, 2.7 mM KCl, 2 mM KH_2PO_4, pH 7.4 at 25°C.

10× NEBuffer 2.1: 100 mM Tris-HCl, 500 mM NaCl, 100 mM $MgCl_2$, 1 mg/ml bovine serum albumin (BSA), pH 7.9 at 25°C.

10× rCutSmart Buffer: 500 mM potassium acetate, 200 mM Tris-acetate, 100 mM magnesium acetate, 1 mg/mL recombinant albumin, pH 7.9 at 25°C.

6× Loading buffer: 0.25% (w/v) bromophenol blue, 40% (w/v) sucrose.

1 × Tris-acetate buffer (TAE): 40 mM Tris, 20 mM acetic acid, 2 mM $Na_2EDTA•2H_2O$, pH 8.0 at 25°C.

1 × Tris-boric acid buffer (TBE): 89 mM Tris, 89 mM boric acid, 100 mM $Na_2EDTA•2H_2O$, pH 8.0 at 25°C.

Preparation of methylated dsDNA

The single-extension PCR was conducted in a 50 μL system with 10 μL template ssDNA (10 μM), 15 μL reverse primer (10 μM) and 25 μL 2 × Phanta Flash Master Mix (Supplied with the kit). The above components were mixed and incubated at 98°C for 40 seconds and 65°C for 10 minutes, followed by incubation at 72°C for 10 minutes for chain extension.

The gel extraction was carried out by a gel extraction kit as follows:

1) The gel slice with the desired DNA bands was cut off under a UV light, placed in a clean centrifuge tube and weighed to obtain an approximate volume at a density of 1 g/mL, and an equal volume of XP2 binding buffer (supplied with the kit) was added and the mixture was incubated at 55°C for complete melting of gel.

2) 700 μL melted gel was transferred to a DNA binding column (supplied with the kit) and the column was inserted into a 2 mL collection tube (supplied with the kit),

followed by centrifuge at 10,000 g for 1 min at room temperature, with the filtrate discarded. This step was repeated in the same binding column and collection tube until all the melted gel was processed.

3) 700 μL ethanol-diluted SPW buffer (supplied with the kit) was added and centrifuged at 15,000 g for 1 minute at room temperature, and the filtrate was discarded. This step is repeated in the same binding column and collection tube until all the solution was processed.

4) The collection tube with obtained product was centrifuged 15,000 g for 2minute at room temperature, with the filtrate discarded and left uncapped for 10 minute until completely dry.

5) The binding column was inserted to a new clean centrifuge tube and 30 μL elution buffer (supplied with the kit) was added to the membrane of the binding column and left at room temperature for 2 minutes, followed by centrifuge at 15,000 g for 1 minute, with the filtrate collected. The obtained filtrate was added to the binding column and centrifuged again, and the target dsDNA with high purity was obtained in the filtrate and stored at 4°C.

The obtained filtrate was further quantified by NanoDrop One and diluted to desired concentrations.

Gel electrophoresis

2% agarose electrophoresis in 1×TAE buffer was employed for the separation of PCR products and 8% non-denaturing poly-acrylamide gel electrophoresis (PAGE) in 1×TBE buffer was employed for the product analysis of HhaI digestion and RPA. The agarose electrophoresis was conducted at 120 V for 60 min and the 8% PAGE was conducted at 120 V for 30 min and the gel was stained by Gel-red for 15 min.

Cell culturing

The cells were cultured in 90% RPMI1640, 10% FBS, 1% PS, with 95% Air and 5% CO_2. The cells were harvested after one week of post-resuscitation culturing, with three batches harvested weekly.

Cell genome extraction

The cells were digested with 1× trypsin and suspended in PBS. Several million cells were added to a 1.5 mL centrifuge tube and centrifuged at 12,000 rpm for 1 minute with the supernatant discarded. The extraction procedure carried out using the DNA extraction kit purchased from Solarbio as follows:

1) 200 μL Solution A (supplied with the kit) was added to the cell containing tube and shaken until thoroughly mixed.

2) 20 μL RNase A (10 mg/mL, supplied with the kit) was added to the suspension and left at 55°C for 15 minutes.

3) 20 μL Proteinase K (10 mg/mL) was added to the suspension and thoroughly mixed for digestion, and left at 55°C for 1 hour to obtain a clear and viscous liquid product.

4) 200 μL of Solution B (supplied with the kit) was added and mixed thoroughly with inversion.

5) 200 μL anhydrous ethanol was added and mixed thoroughly, and transferred to the adsorption column (supplied with the kit), then centrifuged at 12,000 rpm for 1 minute with the supernatant discarded. Insert the column into the collection tube (supplied with the kit).

6) 600 μL ethanol-diluted rinse solution (supplied with the kit) was added to the column and centrifuged at 12,000 rpm for 1 minutes, with the supernatant discarded. Repeat this step with the same column.

7) Centrifuged at 12,000 for 2 minutes and leave open at room temperature to remove residual solution.

8) The column was inserted into a clean 1.5 mL centrifuge tube, and 100 μL of eluent (supplied with the kit, pre-warmed at 65°C) was added to the membrane of the column and incubated at room temperature for 5 minutes, followed by a centrifuge at 12,000 rpm for 2 minutes.

9) The obtained solution was added to the column and centrifuged again to obtain the genome DNA with high quality. The obtained genome DNA was stored at 4°C.

The obtained genomes were quantified by NanoDrop One and adjusted to be consistent before subjected to the detection system.

Nucleic acid sequence involved in this work

Table S1. The nucleic acid sequences involved in this work

Name	Sequence (5’ to 3’)
Methylated DNA	TCTATCATCCAATCACTGCACTTTACACACTATAAATAGAGCAGCTCATGGGCGTA**TTTG**[CG]CTAGTGTTGGGTGTTCCGCTGTGCTGTTTTTCCGTCATGGCTCGCACTAAGCAAACTG
Unmethylated DNA	TCTATCATCCAATCACTGCACTTTACACACTATAAATAGAGCAGCTCATGGGCGTA**TTTG**CGCTAGTGTTGGGTGTTCCGCTGTGCTGTTTTTCCGTCATGGCTCGCACTAAGCAAACTG
Forward	TCTATCATCCAATCACTGCACTTTACACACT
Reverse	CAGTTTGCTTAGTGCGAGCCATGACGGAAA
crRNA	UAAUUUCUACUAAGUGUAGAUCGCUAGUGUUGGGUGUUCCGCU
F-Q probe	6-FAM-TTTTT-BHQ1

Note: The bases in brackets represent methylated bases. PAM are written in bold, and the underlined nucleotides represent cleavage sites for HhaI.

2. Optimization of HGRC

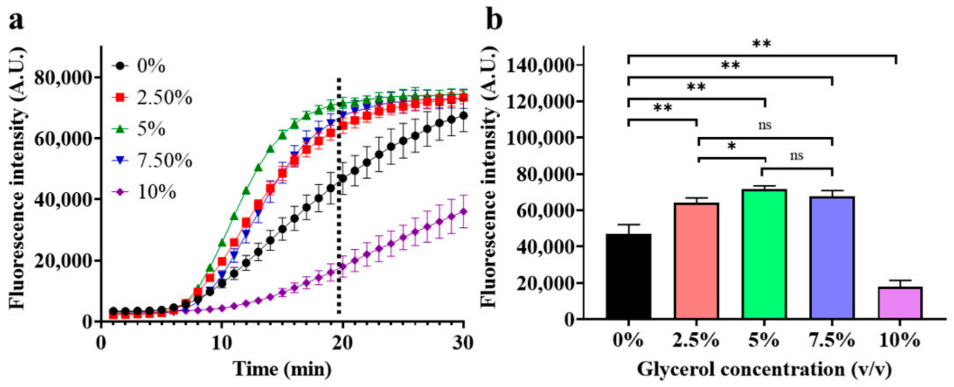

Figure S1. Glycerol concentration optimization on target methylation at concentration of 1 pM. **(a).** Real-time fluorescence intensity of each group. **(b).** Fluorescence intensity after a 20-minute reaction. (Mean ± s.d., n = 3 technical replicates, variance was calculated using Student's t-test, * represents $p < 0.05$, ** represents $p < 0.01$, ns

represents non-significant.)

As depicted in Figure S1, for target at a concentration of 1 pM, the reaction process is accelerated due to the increased target concentration, and the reaction is almost complete after 25 min. It can also be observed that during the reaction, the fluorescence generation rate is significantly larger in groups with glycerol concentration of 2.5-7.5% than in the group with 0 glycerol addition. As shown in Figure S1B, taking the fluorescence intensity at the 20th minute as an example, the fluorescence intensity of the 5% group is significantly higher than that of the other groups, which also indicates that the 5% is the optimal glycerol concentration for this system.

3. Sensitivity

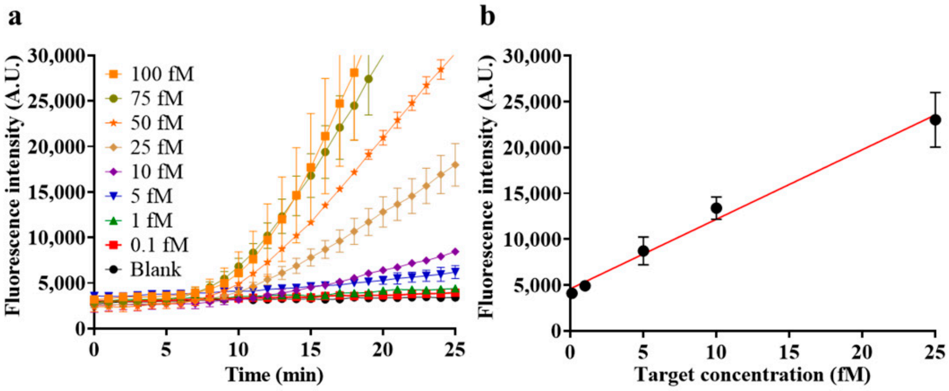

Figure S2. Zoomed-in view of targets with lower concentrations. **(a).** Real-time fluorescence intensity plot. **(b).** Endpoint fluorescence intensity plot. (Mean ± s.d., n = 3 technical replicates.)

As depicted in Figure S2, As can be seen in Figure S2, the fluorescence intensity and concentration still maintain a good linear relationship when the concentration become lower.

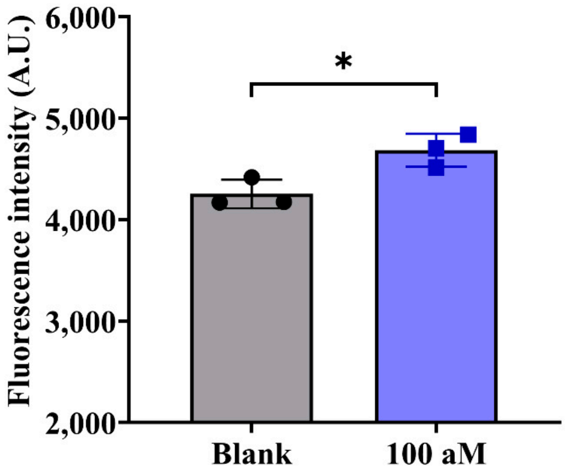

Figure S3. Comparison between detecting 100 aM target and blank control. (Mean ± s.d., n = 3 technical replicates. Variance was calculated using student's t-test, * represents $p<0.01$.)

As depicted in Figure S3, the HGRC is capable of distinguishing target methylation as low as 0.1 fM from blank control and the LOD is defined as 100 aM.
